# Determinants of Protein Abundance and Translation Efficiency in S. cerevisiae

**DOI:** 10.1371/journal.pcbi.0030248

**Published:** 2007-12-21

**Authors:** Tamir Tuller, Martin Kupiec, Eytan Ruppin

**Affiliations:** 1 School of Computer Science, Tel Aviv University, Tel Aviv, Israel; 2 Department of Molecular Microbiology and Biotechnology, Tel Aviv University, Tel Aviv, Israel; 3 School of Medicine, Tel Aviv University, Tel Aviv, Israel; Yale University, United States of America

## Abstract

The translation efficiency of most *Saccharomyces cerevisiae* genes remains fairly constant across poor and rich growth media. This observation has led us to revisit the available data and to examine the potential utility of a protein abundance predictor in reinterpreting existing mRNA expression data. Our predictor is based on large-scale data of mRNA levels*,* the tRNA adaptation index, and the evolutionary rate. It attains a correlation of 0.76 with experimentally determined protein abundance levels on unseen data and successfully cross-predicts protein abundance levels in another yeast species (Schizosaccharomyces pombe)*.* The predicted abundance levels of proteins in known S. cerevisiae complexes, and of interacting proteins, are significantly more coherent than their corresponding mRNA expression levels. Analysis of gene expression measurement experiments using the predicted protein abundance levels yields new insights that are not readily discernable when clustering the corresponding mRNA expression levels. Comparing protein abundance levels across poor and rich media, we find a general trend for homeostatic regulation where transcription and translation change in a reciprocal manner. This phenomenon is more prominent near origins of replications. Our analysis shows that in parallel to the adaptation occurring at the tRNA level via the codon bias, proteins do undergo a complementary adaptation at the amino acid level to further increase their abundance.

## Author Summary

DNA microarrays measuring gene expression levels have been a mainstay of systems biology research, but since proteins are more direct mediators of cellular processes, protein abundance levels are likely to be a better indicator of the cellular state. However, as proteomic measurements are still lagging behind gene expression measurements, there has been considerable effort in recent years to study the correlations between gene expression (and a plethora of protein characteristics) and protein abundance. Addressing this challenge, the current study is one of the first to introduce a predictor for protein abundance levels that is tested and validated on unseen data using all currently available large-scale proteomic data. The utility of this predictor is shown via a comprehensive set of tests and applications, including improved functional coherency of complexes and interacting proteins, better fit with gene phenotypic data, cross-species prediction of protein abundance, and most importantly, the reinterpretation of existing gene expression microarray data. Finally, our revisit and analysis of the existing large-scale proteomic data reveals new key insights concerning the regulation of translation efficiency and its evolution. Overall, a solid protein abundance prediction tool is invaluable for advancing our understanding of cellular processes; this study presents a further step in this direction.

## Introduction

DNA microarrays are now commonly used to measure the expression levels of large numbers of genes simultaneously [1]. Since proteins are the direct mediators of cellular processes, the abundance level of each protein is likely to be a better indicator of the cellular state than its corresponding mRNA expression level. However, genome-wide technologies to detect protein abundance are still lagging behind those that measure mRNA, and only few studies that measure protein abundance on a large scale are currently available [2–6].

The relationship between mRNA and protein abundance levels has been studied by several groups. Genes with similar mRNA levels may have very different protein abundance levels [7]. Yet, the correlation between protein and mRNA abundance after a log-transform was shown to be quite high [8]. A more recent study, combining three technologies for measuring mRNA expression, has yielded correlation levels of about 0.7 with protein abundance [9]. Several studies have aimed at correlating protein abundance to various other features of proteins, such as their codon bias, molecular weight, stop codon identity, and more [3,4,10,11] These investigations and other previous proteomic studies [12–14] were usually based on small- to medium-scale measurements.

The current study revisits these issues and presents a comprehensive investigation of the relationship between factors that influence protein abundance and the associated protein levels. We begin by constructing a predictor for protein abundance levels, which, in contrast to previous studies, is tested and validated on unseen data (see Methods). To this end, we rely on two large-scale protein abundance datasets [2,5]. Overall, to our knowledge this is the first time that the whole body of data currently available is collated and analyzed to this aim, and we obtain a predictor with a correlation of 0.76 with experimentally determined abundance levels. Applying the resulting predictor to pertaining mRNA expression data testifies to its utility. Our analysis provides new key insights concerning the regulation of translation efficiency and its evolution.

## Results

**Figure 1:**
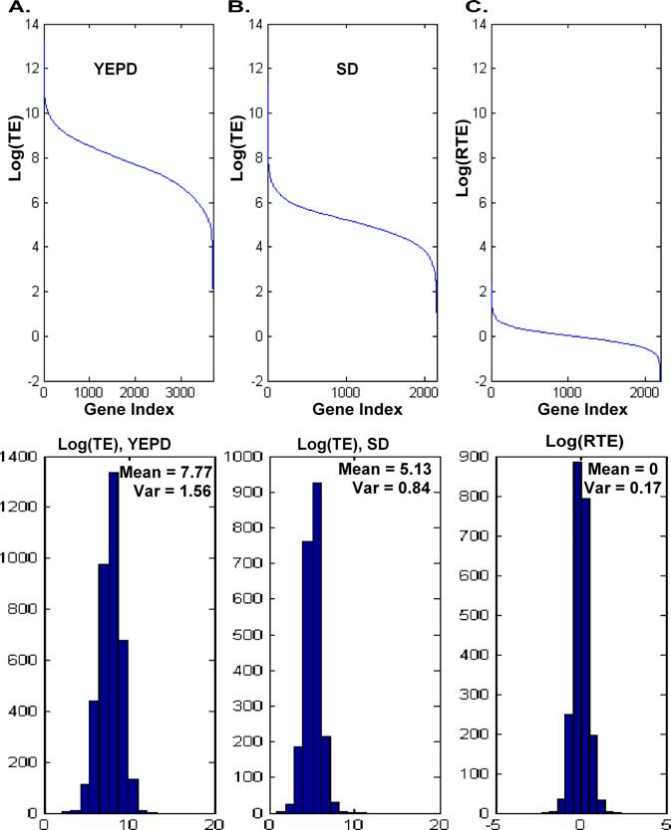
Distribution of TE and RTE in S. cerevisiae (A) Top: S. cerevisiae genes sorted by their TE (log scale) in YEPD (rich) medium. A large variability of TE values (more than six orders of magnitude) is observed. Bottom: histogram, mean, and variance of TE in YEPD. (B) Top: S. cerevisiae genes sorted by their TE (log scale) in SD (poor) medium. A similar large variability of TE values is seen. Bottom: histogram, mean, and variance of TE in SD. (C) Top: S. cerevisiae genes sorted by the log-ratio of their TEs [RTE = (*p_SD_*/*m_SD_*)/(*p_YEPD_*/*m_YEPD_*)] in SD versus YEPD (log scale). A total of 91% of the genes have an RTE value between 0.5 and 2. Bottom: histogram, mean, and variance of RTE.

Genome-wide studies have measured mRNA and protein levels in the yeast Saccharomyces cerevisiae growing either in rich medium (yeast extract, peptone, and dextrose [YEPD]) or on poor, defined medium (synthetic dextrose [SD]) [2,3,5]. When protein abundance is compared to the corresponding mRNA levels in a given medium, the translation efficiency (TE), i.e., the ratio between protein abundance and mRNA levels, exhibits a large variability among genes (spanning across six orders of magnitude; Figure 1A and 1B). However, when the TEs of a given protein are compared across the two different growth conditions, notably very little variation is observed (Figure 1C): the ratios between the TEs of most proteins in the two conditions are close to 1, with >90% of the proteins showing a ratio between 0.5 and 2. This observation, albeit currently limited to the two types of media for which genome-wide data are available, suggests that the efficiency of translation per mRNA molecule of many genes may be largely invariable under different conditions. This fairly constant TE of yeast genes has motivated us to create a large-scale predictor of protein abundance, with the aim of studying its utility for inferring protein abundance levels across different conditions.

The simplest predictor we studied is a linear one based on mRNA expression levels. Training this predictor on a randomly selected subset of the full complement of yeast mRNA and protein levels yields a Spearman rank correlation coefficient of *r_s_* = 0.55 on held-out test data (the protein abundance was from [2] and mRNA levels were from [15]; see Methods). To improve the prediction accuracy, we examined the potential utility of combining 32 additional protein attributes into a multivariable linear predictor, some of which have been previously shown to have predictive value (Table S1). A greedy feature selection algorithm identified two useful protein attributes, while the inclusion of all other features resulted in a marginal and insignificant improvement in the performance of the linear, mRNA-based predictor. Performing the prediction by a support vector machine (SVM) using a variety of nonlinear kernels did not improve the results (Methods).

**Figure 2:**
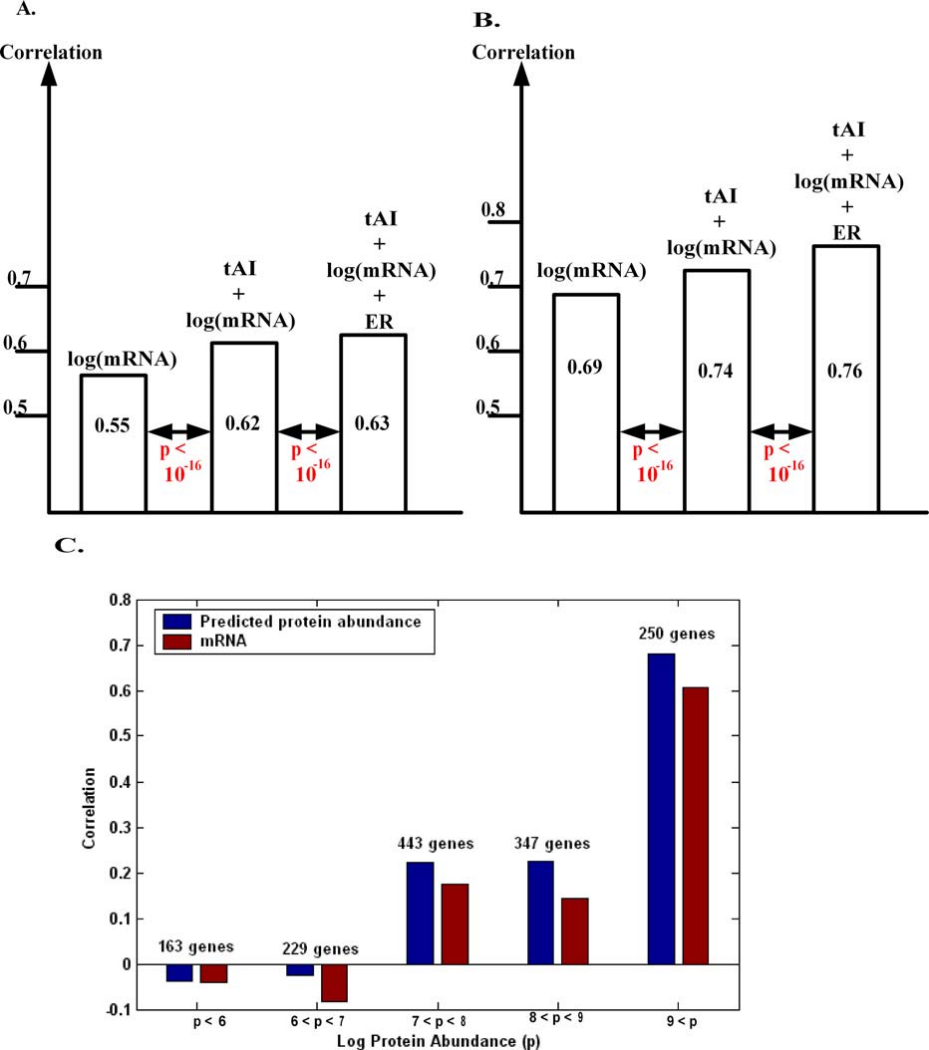
Performances of the Linear Predictor of (log) Protein Abundance (A) The accuracy of various linear predictors of (log) protein abundance, measured by the Spearman rank correlation coefficient over a held-out test set, using a single data source of protein abundance [2] and mRNA levels [15]. ER values are from [19], and tAI data are taken from [20]. The numbers below the arrows denote the *t*-test *p*-values for checking the null hypothesis that the predictor with the new added feature has identical performance to its predecessor (see Methods). The final predictor for protein abundance (PA) is log(*PA*) = 3.97 + 0.4 × log(*mRNA*) + 10.34 × *tAI* − 3.35 × *ER*. (B) Accuracy of various linear predictors, in the case where protein and mRNA levels are generated by averaging measurements from at least two data sources. The final predictor for protein abundance obtained in this case is log(*PA*) = 3.47 + 0.63 × log(*mRNA*) + 10.89 × *tAI* − 2.923 × *ER*. (C) The Spearman correlations (*y*-axis) of predicted protein abundance (mRNA) with measured protein abundance levels, binned at various levels of protein abundance *p* (*x*-axis, natural log). All the correlations are higher and significant in the case of predicted protein abundance (*p* < 2 × 10^−5^), except for the lowest bin log(*p*) < 7.

The two protein features yielding a significant improvement in prediction accuracy were the tRNA adaptation index (tAI) [16,17], and the evolutionary rate (ER) [18,19]. tAI is based on the synonymous codon usage bias and gene copy number of different tRNAs and is related to the codon adaptation index (CAI) [16,17]. ER measures the rate of evolution of a protein by comparing its orthologs across related species [18,19]. These two features have been shown previously to be correlated with protein abundance levels [18,20]. Combining tAI with mRNA levels increases the prediction accuracy from the levels of *r_s_* = 0.55 obtained using mRNA levels alone to a Spearman rank correlation coefficient of *r_s_* = 0.61 on the same dataset as above. Adding evolutionary rate values increases the correlation to 0.63. The incremental improvement of consecutively adding these two features to the basic linear regression protein abundance predictor is statistically significant (Figure 2 and Methods).

Large-scale measurements of mRNA and protein levels tend to be noisy. Thus, in the (yet rare) cases where several independent measurements of mRNA and protein levels at the same conditions are available, they can be used to reduce potential individual measurement biases by pooling them together [9] (the correlation between two proteomic datasets generated by two different techniques and in different labs are between *r_s_* = 0.6 and *r_s_* = 0.8; see Text S1). We thus averaged mRNA and protein abundance results obtained with different technologies (see Methods for the description of the pertaining datasets used to this end). This results in a further notable improvement of prediction accuracy (*r_s_* = 0.76; Figure 2), suggesting that a considerable fraction of the variability in the datasets is due to experimental measurement errors (the improvement of the correlations observed upon averaging can also be due to the blurring of the effects of different posttranscriptional regulation processes taking place in the different conditions in which the measurements were done [temperature, strains, media, technique], but since we averaged over relatively similar conditions, we expect this effect to be relatively minor). In the following investigations reported in this paper, multiple independent measurements at the same conditions were not available, and the results reported are hence without pooling and averaging the data.

Examining the performance of our YEPD-trained predictor on a new unseen dataset of 238 genes whose protein abundance levels were measured under very different conditions (exposure to pheromone [13]) resulted in a high correlation of *r_s_* = 0.69. The correlation between mRNA levels solely and protein abundance levels was 0.62, in comparison. The standard deviation of 1,000 cross-validation runs of the predictor was 0.016, and the improvement compared to mRNA-based prediction was significant, with *p* < 10^−16^. Further information on the predictors' performance on specific Gene Ontology (GO) annotation gene sets is provided in Table S2. This table also shows that the predictor improves the prediction of protein abundance levels (compared to mRNA levels) in 92% of the GO annotation categories. Our predictor obtains higher correlations with protein abundance levels than using mRNA alone across numerous ranges of protein abundance; however, this correlation is not statistically significant in the lowest protein abundance range (Figure 2C).

Using our multivariate linear predictor, expression of genes whose products are members of the same complex (according to SGD [21]) exhibits significantly higher coherency than when calculated from their corresponding mRNA levels. Table 1 displays the pertaining Spearman rank correlation coefficients for pairs of genes that are part of the same complex. For the cases of experimentally determined and predicted protein abundance levels, we also computed the partial correlations after controlling for the effect of mRNA expression levels (Methods). A similar, but weaker trend is also observed when examining the abundance coherency of protein pairs that exhibit a protein–protein interaction (Text S2). These results indicate that our prediction approach is likely to be more appropriate for proteins in large macromolecular complexes than for proteins involved in signaling and transcriptional control, since the latter are heavily posttranslationally modified. This notion is further supported by noting that in the highest protein abundance bin (Figure 2C), there are 26 genes that are related to the “Ribosome” GO category, providing a hyper-geometric enrichment of *p* < 4.2 × 10^−4^.

**Table 1:**
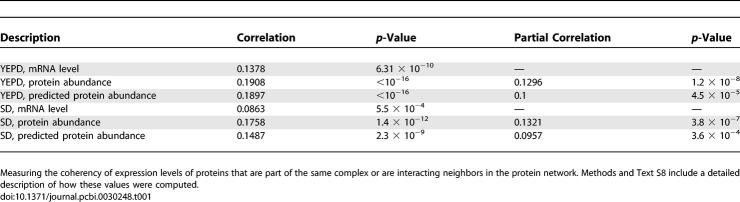
The Spearman Rank Correlation Coefficients and Partial Spearman Correlations between mRNA, Protein Abundance, and Predicted Protein Abundance Levels in YEPD and SD for Gene Pairs That Are Part of the Same Complex

Given the observation that the TE of most proteins is fairly similar across the two different conditions analyzed, we examined the utility of the protein abundance predictor in interpreting the results of two yeast mRNA gene expression datasets, obtained under a variety of environmental conditions (see Text S3). The first dataset investigated the yeast response to low-shear modeled microgravity. It included 12 different conditions (six under low-shear and six controls) [22]. To analyze this dataset, we clustered and bi-clustered the genes in the microarray data in accordance with the mRNA expression patterns, in a conventional manner. In parallel, we used our predictor to generate predicted protein abundance levels from the expression levels, and repeated the clustering and bi-clustering process on the resulting protein abundance data. We then compared the resulting cluster sets with respect to their functional enrichment in GO annotations (Methods). We performed a similar analysis on a gene expression dataset consisting of 36 timepoints taken from yeast cells growing in continuous, nutrient-limited conditions [23] (the first dataset includes gene expression measurements of a system that is close to equilibrium, while the second includes gene expression measurements of a system in a transient state; see Text S4).

Table 2 shows that the use of the predicted protein abundance values in these datasets results in a significant increase in the percentage of clusters that exhibit enrichment for specific GO terms (for comparison, random predictors significantly deteriorate the clustering enrichment scores; see Text S5). In the case of Sheehan's data [22], the protein abundance predictor improved both the separation and the homogeneity. In the case of Tu's data [23], the homogeneity improved while the separation score deteriorated (Table 2). A closer analysis provides evidence for the advantage of using the predictor: in the first dataset, a new bi-cluster is detected (cluster 4) in the protein abundance analysis that does not appear in the mRNA level analysis. This bi-cluster spans over 11 of the 12 conditions and is enriched with many GO annotations (mainly related to metabolism; Table S4). Similarly, in the second dataset, cluster 7 in the predicted protein abundance analysis is a novel group that does not appear when analyzing mRNA levels. This cluster shows a striking periodic expression that coincides with the respiratory bursts observed under continuous nutrient-limited conditions [23]. Thus, using predicted protein abundance levels, a simple conventional clustering method suffices to reveal novel central clusters that were not apparent in the original study at the mRNA expression level. Tables S3, S4, S5, and S6 provide a detailed analysis (list of clusters, bi-clusters, and GO enrichments) for the two datasets.

**Table 2:**
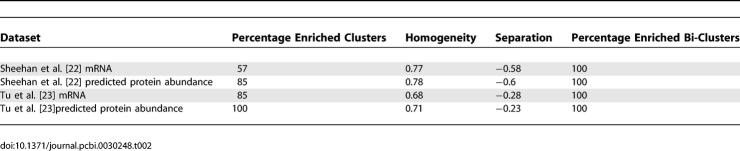
The Percentage of GO-Enriched Clusters and the Percentage of GO-Enriched Bi-Clusters Obtained by Analyzing mRNA Levels or Predicted Protein Abundance Levels in Two Gene Expression Datasets, and the Total Homogeneity and Separation Scores for the Clustering Results

We used our protein abundance predictor to reanalyze the intriguing results reported by [24], showing that only a very small fraction of the genes whose expression is significantly elevated under a specific condition actually cause a significant decrease in fitness when deleted. Overall, we find that the fraction of expressed genes that lead to a significant reduction in fitness when deleted is 2-fold to 3-fold higher than the corresponding fraction reported using mRNA levels (e.g., 2.9% versus 0.76% in the case of yeast cells responding to 1.5 M sorbitol, and 13.2% versus 6.4% in the case of 1 M NaCl). Although the absolute fraction of genes accounted for still remains small, the relative increase observed by using the predictor is substantial.

Finally, we tested our predictor's ability to correctly estimate protein abundance levels from mRNA expression data in a different organism, *Schizosaccharomyces pombe.* To this end, we used mRNA and protein data from a recent genome-wide study that reported a Spearman rank correlation coefficient of 0.61 between the two measurements [25]. Focusing on a subset of S. pombe genes that have an ortholog in *S. cerevisiae,* the Spearman rank correlation of the predicted protein levels with actual protein abundance measurements was 0.675. Notably, for the same subset of genes, the Spearman rank correlation between the protein abundance and mRNA levels of S. pombe was only 0.629 (and the rank correlation between the mRNA levels of the two organisms was 0.48). These results are quite remarkable, since the predictor used to predict protein abundance in S. pombe was based on the ER and tAI values of the corresponding orthologs in *S. cerevisiae.*

**Figure 3:**
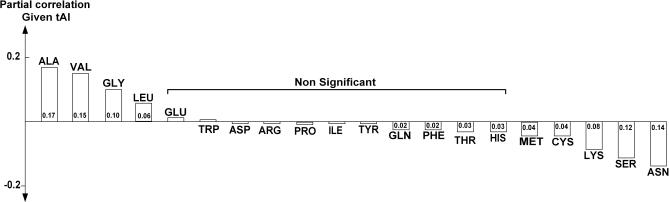
Partial Correlations between the Frequencies of Amino Acids Composing a Protein and Its Abundance Level (after Controlling for the Effect of tAI)

Like previous studies [4,26], we have also found a significant correlation between the abundance of a particular protein and the frequency of certain amino acids composing it, the most prominent being alanine and valine (positive correlation), and serine and aspargine (negative correlation; Figure S1). This observation has been previously attributed to the different values of the tAI (or the CAI) of these amino acids, which can modulate translation efficiency [16,17]. However, we find that even after controlling for the effect of their different tAIs, the frequency of these amino acids remains significantly correlated with protein abundance, and their frequency at abundant proteins remains highly significant (see partial correlations reported in Figure 3, and similar results after controlling for CAI in Figure S2). The Spearman rank correlation of amino acid frequencies and protein abundance remains significant even after additionally controlling for the effect of mRNA expression levels (Table S7). This finding suggests that in parallel to the adaptation occurring at the tRNA level via the codon bias [27,28], proteins do undergo a complementary adaptation at the amino acid level via amino acid substitution to further increase their protein abundance. The small, neutral, and nonpolar amino acid alanine is probably ideally suited for this putative substitute role, given its known neutral effect on protein stability [29]. Both alanine and valine are present at relatively high concentrations within the yeast cell, and their corresponding acyl-tRNA synthases are also expressed at high levels (Table S8), aiding in their efficient incorporation during transcription (however, adding frequencies of amino acids to our predictor did not improve its performance significantly; see Text S6).

The recent direct measurement of absolute protein levels under two distinct growth conditions [5] enabled us to compare the ratio between the translation efficiency observed in cells grown on poor medium versus the one observed in rich medium, i.e., the relative TE (RTE; (*p*/*m*)*_SD_*/(*p*/*m*)*_YEPD_*). There is a significant negative correlation (−0.213; *p* < 10^−50^) between the RTE and the change in transcription levels between the two growth conditions. Even when focusing only on genes that change their protein abundance between the two conditions in a considerable manner (protein abundance ratio > 1.4 or < 1/1.4), the resulting negative correlation remains significant (*r* = −0.08; *p* = 0.018). This may suggest that there is a global homeostasis between transcription and translation, with a tendency to increase translation when transcription decreases, and vice versa. The average RTE is 1.091 (about half the genes, 1,072 out of 2,204, have RTE > 1). Since the relative decrease of the ribosomal protein abundance (*p_SD_*/*p_YEPD_* = 0.88) is higher than the total relative decrease of mRNA levels (*m_SD_*/*m_YEPD_* = 0.98), the number of ribosomes per mRNA is lower in SD. Thus, the findings of average RTE > 1 are probably due to lower protein degradation rates or other causes of higher translation rates in SD, rather than increased ribosomes per mRNA levels (Figure S3 depicts the mean RTE levels of different GO annotation groups; Text S7 displays the variance in protein abundance levels in the two growth conditions).

While the large majority of the genes have RTE levels ranging between 0.5 and 2 (Figure 1B), two sets have extreme RTE values, one with RTE > 2.5 (48 genes), and the other with RTE < 0.45 (65 genes; Tables S9 and S10). The distribution of mRNA and protein abundance levels of genes within each of these groups is similar to that of the rest of the genes (see Figure S4A and S4B), and extreme ratios of protein abundance or mRNA levels do not necessarily imply extreme RTE values (see Figure S4C). Interestingly, our predictor obtains more significant improvement in the correlations with actual protein abundance levels on genes with extreme RTEs (see Figure S4D). In contrast to the inverse (homeostatic) relation observed in general, the set with extremely high RTE also exhibits extremely high *m_SD_*/*m_YEPD_* ratios (an average mRNA ratio of 5.35, 14 times the general average). This indicates that the extreme RTE values reflect the fact that the cell is making a concerted effort to maintain their protein abundance levels at the extreme levels needed. By the same token, the mean mRNA ratio for the set with extremely low RTE is 0.36, somewhat below the total average.

**Figure 4:**
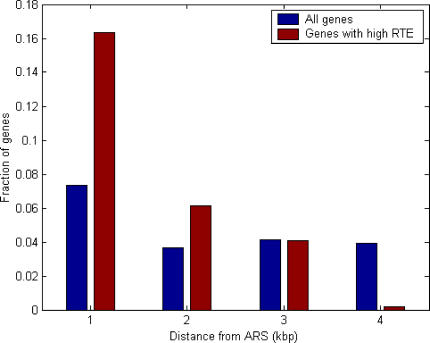
The Distribution of Genes with High RTEs at Different Distances from Origins of Replication The distribution of genes with high RTE (RTE > 2.5), and distribution of all genes at different distances from origins of replication. The number of genes with high RTE is 49; the total number of genes studied is 2,200. The number of genes with high RTE that are located within 1 kbp from an ARS is statistically significant using a hyper-geometric text (*p* < 0.05).

The group of genes exhibiting extremely high RTE levels is enriched for mitochondrial genes (21/48 are mitochondrial genes; chi-square *p* = 10^−16^), with many of these genes being related to mitochondrial biosynthesis and metabolism. Thus, the increase in the level of mitochondrial proteins, reflecting the need for higher-yield energy production in poor growth conditions, is achieved mainly by boosting translation efficiency. Interestingly, the high RTE group is also enriched with genes that map very close to origins of replication (autonomously replicating sequence [ARS]), including four genes abutting at the origin of replication (out of a total of 24 genes with a similar location in the yeast genome, providing a chi-square *p* = 1.1 × 10^−6^), and twice the expected number of genes located within 1 kbp from an ARS (*p* < 0.05; see Figure 4). A possible explanation for this intriguing connection is that the replication machinery, when binding to origins of replication, attenuates transcription, either by steric hindrance or by competition for DNA binding [30]. This interference is then compensated in turn by higher translation efficiency and a more flexible regulation of translation, as reflected by its high RTE levels. Indeed, the average *m_SD_* /*m_YEPD_* ratios of genes that have extremely high RTE and that are less than 1 kb from an ARS is only 0.8. One putative mechanism that may underlie this intriguing phenomenon is that certain proteins that participate in replication and transcription (e.g., Rap1 and Abs1) could be incorporated into the mRNA, exported from the nucleus, and differentially affect the rate of translation at the ribosome. Similar mechanisms have been suggested for the activity of proteins such as Yra1, Sub2, and the THO complex, which affect transcription, splicing efficiency, and nuclear export [31].

## Discussion

The availability of whole-genome measurements of protein abundance provides a unique opportunity to analyze the forces that affect protein translation and abundance. Combining several protein features yields a predictor of protein abundance that can serve as a useful tool for analyzing gene expression measurements. Our results indicate that highly expressed proteins undergo adaptation at the amino acid level, and that proximity to an origin of replication enhances the efficiency of translation.

Translation efficiency is determined by invariant, condition-independent factors such as the amino acid and codon composition of the protein and the availability of the different tRNAs. It is also modulated by dynamic factors such as ribosome occupancy and ribosome density (determining the total number of ribosomes per mRNA), which are dependent on environmental clues [10]. Assuming that TE is constant to a first approximation for most genes (as its levels across poor and rich media testifies), this study has focused on the first group of factors, and has shown the utility of such a predictor in interpreting biological data. We anticipate that as information gradually accumulates concerning the second group of factors, more accurate protein abundance predictors will emerge that can incorporate information on posttranscriptional regulation [32–34]. Recent work has suggested that transcription factors and signaling genes tend to be posttranscriptionally regulated [32]. Indeed, a large proportion of the genes with extreme RTE levels belong to these two categories (see Tables S9 and S13). However, not all genes regulated at the posttranscriptional level exhibit extreme RTE values: a recent genome-wide study in yeast has identified 16 genes with extreme TE levels, presumably regulated posttranscriptionally [9]. Examination of the RTE levels of these genes reveals that only one has extreme RTE levels (*MET6,* with RTE = 0.47); the rest have RTE levels between 0.93 and 1.38 (see Table S13). Finally, protein degradation and turnover are obviously important modulators of protein abundance, and should be considered in future predictors as pertaining reliable data accumulates. That said, it is interesting and encouraging to see how far one can go in predicting protein abundance levels even without this information.

An important corollary of our work is that gene expression results obtained with DNA microarray technology may in some cases be misleading. For example, Tables S11 and S12 include a subset of genes that exhibit inversely correlated regulatory trends at the transcription versus the translation level. An increase in mRNA expression levels of a particular gene does not necessarily mean a higher level of its protein. The corresponding protein abundance could not be differentially expressed or could even be differentially expressed but in the opposite direction. As Tables S11 and S12 include about 5% of the yeast genes, this type of error may be nonnegligible at times. Our predictor cannot solve this problem; its solution will probably require much larger biological datasets than those currently available.

We demonstrated that our predictor (which is based on S. cerevisiae) can be used to successfully predict protein abundance levels in a different organism (S. pombe), which has an evolutionary distance of 350–1,000 million y from S. cerevisiae [35]. It will be interesting to examine the effect that evolutionary distance may have on determining the “transferability” of protein predictors across species. However, answers to this question will need to wait until protein abundance data of additional organisms becomes available.

Building on the existing large-scale protein abundance data, this study has shown that a predictor of protein abundance levels can improve the interpretation of gene expression measurements and provide new insights into the regulation and evolution of protein translation. The utility of such a tool should be further enhanced as our understanding of the determinants affecting protein abundance and translation improves and the pertaining data continues to accumulate.

## Methods

### Generating a predictor of protein abundance.

For training the predictors, we used all the genes whose required features (mRNA measurements, protein abundance, ER, tAI) were available. The series of linear predictors studied were generated using a linear regressor and using the following cross-validation procedure: (1) randomly choose 80% of the genes (training set) and use them for generating a linear predictor; (2) use the resulting predictor for predicting the protein abundance of the remaining 20% of the genes (test set); and (3) for the genes in the test set, calculate the Spearman rank correlation coefficient between the predicted and experimentally measured protein abundance values.

This cross-validation procedure is repeated 10^5^ times, and the mean of the Spearman rank correlation coefficient (computed in step 3) is the predictor accuracy reported in the main text.

As reported in the main text, we generated a sequence of linear predictors of protein abundance, each time adding the most informative feature in a greedy manner. During this process, we checked if the resulting incremental improvement in prediction performance is statistically significant by performing a *t*-test, comparing the distribution of Spearman rank correlation coefficients obtained by each predictor over the 10^5^ cross-validation runs. Note that in the case of a multivariate linear predictor, this cross-validation procedure may lead to similar prediction accuracy values as those obtained by training a multivariate regressor on the whole dataset. However, in the general scope of nonlinear predictors investigated in this study, the cross-validation prediction scenario used is conceptually different from a multivariate regression, and the results obtained significantly differ.

Going beyond a linear predictor, we used two implementations of SVMs, SVM-light [36] and Partek (Partek Software, http://www.partek.com), and examined radial, polynomial, and sigmoid kernels. The initial set of features included all the 32 features described in Table S1, and we also examined various forward and backward algorithms for feature selection. Quite surprisingly, none of these SVM predictors gave a significant increase in prediction performance compared to the best linear predictor reported upon in the main text. In constructing the predictors we used the following data sources.

*Protein abundance and mRNA expression data.* We analyzed four protein abundance datasets: (1) a dataset generated by merging (with the appropriate normalization) protein abundance data from numerous small-scale datasets [3]; (2) a large-scale measurement of protein abundance in yeast (normal log phase) [2]; and (3) protein abundance large-scale measurements by [5] in two different growth media conditions (YEPD and SD). We analyzed two major mRNA expression datasets: (1) one generated by combining 36 microarray datasets (wild-type yeast grown in YEPD without any stress) [10]; and (2) an mRNA measurement of wild-type yeast grown in YEPD [21].

The dataset of [5] also includes the ratio (but not the absolute values) between the mRNA levels in the two conditions (SD and YEPD), *m_SD_* /*m_YEPD_*. This information, combined with the protein abundance measurements in these two conditions, enabled us to compute the RTEs across growth conditions. Combined with the absolute mRNA measurements from [2], it was used to calculate the absolute mRNA levels in SD.

For computing mean protein abundance levels in constructing the pooled-data predictor, we averaged at least two of three measurements reported in [2,5,8]. For computing mean mRNA abundance levels to this construction, we averaged at least two of three measurements reported in [21,37,38]. The averaging was done following the procedure described in [9].

*Sources of additional data.* Protein half-life measurements were obtained from Belle et al. [39]. The protein properties examined in the construction of the protein abundance predictor (properties 1–28 in Table S1) were obtained from the *Saccharomyces* genome database [21]. The tAI data were downloaded from [20]. Evolutionary rates of proteins were taken from Wall et al. [19]. The mRNA gene expression data, protein abundance data, and list of 447 relevant orthologous genes needed for testing the predictor performance on S. pombe were from [25]. Relative protein abundance and mRNA levels after exposure to pheromone were downloaded from [13].

### Clustering, bi-clustering, and GO enrichment analysis of mRNA and predicted protein abundance levels.

We used two mRNA gene expression datasets that were generated by the same technology as that used for training the predictor. The two datasets are measurements by affymetrix GeneChip, and were downloaded from National Center for Biotechnology Information (NCBI) Gene Expression Omnibus (GEO; http://www.ncbi.nlm.nih.gov/entrez/query.fcgi?db=gds). The first dataset includes the 12 samples from [22]. The second dataset includes the 36 samples from [23]. Clustering and bi-clustering was performed by using the Expander program [40]. We used CLICK for clustering and SAMBA for bi-clustering. Gene enrichment was computed using the GO categories of [21] (by computing the hyper-geometric probability of seeing at least *x* number of genes out of the total n genes in the cluster/bi-cluster annotated to a particular GO term, given the proportion of genes in the whole genome that are annotated to that GO term), examining the three ontologies of molecular function, biological process, and cellular components. The resulting enrichments were filtered by false discovery rate (FDR) to correct for multiple testing [41].

### Measuring the coherency of expression levels of proteins that are part of the same complex or are interacting neighbors in the protein network.

Protein complex data were downloaded from [21]. We measured coherency of mRNA levels, protein abundance, and predicted protein abundance of genes that are part of the same complex (in SD and YEPD) by the following steps: (1) we listed all pairs of genes in the dataset which are both comembers in one of the complexes; (2) for each case (mRNA levels, protein abundance, and predicted protein abundance), we generated two vectors, *u* and *v*, such that *u*(*i*) and *v*(*i*) denote a pair of proteins that are part of the same complex; we calculated the Spearman rank correlation coefficient between the two vectors (*u* and *v*); and we compared the resulting correlation to the correlations between pairs of vectors with the same length that include measurements of randomly selected pairs of genes.

For predicting protein abundance, we used a predictor that was trained on a different dataset (i.e., the predictor used for YEPD was trained on the SD measurements and vice versa; training the predictor on the same dataset gives an even better result, so we wanted to demonstrate that the results are significantly good even if the trained set and the test set are different.). The computation of the pertaining partial correlations and their associated *p*-values are described in Text S8.

For computing the coherency of expression/abundance of neighboring proteins in the protein interaction network, we used the yeast protein interaction network from the work of [42].

We used a similar procedure to that used to compute the complexes' coherency, but this time *u* and *v* are composed of protein pairs that are adjacent in the protein interaction network.

### Comparing mRNA expression profiling and fitness profiling.

For comparing the number of genes that exhibits both an increase in expression levels (mRNA levels and predicted protein abundance) and a significant decrease in fitness when adding NaCl or sorbitol, we used the mRNA levels from [43] and fitness profiling from [24]. For each of the two cases (mRNA levels and predicted protein abundance), we used five measurements of expression levels and four measurements of fitness. We focused on the set of genes for which we had all the predictor's features. In the case of fitness profiling, a gene was considered “significant” if it had significant value (as defined in [24]) in at least one of the four fitness measurements. In both cases of protein abundance or mRNA expression levels, a gene was considered significant if it exhibited a log ratio of at least 0.25 in one of the five measurements.

## Supporting Information

Figure S1 Variables That Have Significant Correlation and Partial Correlation with Protein Abundance, TE, and RTE (A) Variables that have significant correlation with protein abundance, TE, and RTE.
(B) Variables with significant correlation with protein abundance given mRNA, given CAI, and given mRNA and CAI. The full names and the description of each variable appear in Table S1. The correlation with amino acid distribution at the C and N terminus was substantially less significant than the general correlations of amino acid distribution (it was not significant for most of the amino acids).
(82 KB DOC)

Figure S2 Partial Correlations of Amino Acid Frequencies and Protein Abundance after Removing the Effect of CAI (53 KB DOC)

Figure S3 The Average RTE of GO Annotation Groups The average RTE of each GO annotation group for the three ontologies (molecular function, cellular component, and biological process).
(71 KB DOC)

Figure S4 mRNA Levels, Protein Abundance, mRNA Ratio, Protein Abundance Ratio, and Correlation with Protein Abundance of mRNA and Predicted Protein Abundance of Genes with Extreme RTE (A) mRNA levels and protein abundance of genes with RTE > 2.5 (blue), RTE < 0.45 (red), and the rest of the genes (yellow) in YEPD.
(B) mRNA levels and protein abundance of genes with RTE > 2.5 (blue), RTE < 0.45 (red), and the rest of the genes (yellow) in SD.
(C) mRNA ratio (*m_SD_*/*m_YEPD_*) levels and protein abundance ratio (*p_SD_*/*p_YEPD_*) of genes with RTE > 2.5 (blue), RTE < 0.45 (red), and the rest of the genes (yellow).
(D) Correlation with protein abundance of mRNA and predicted protein abundance for genes with modest RTE (0.5 < RTE < 2), and for genes with extreme RTE (RTE < 0.5 and RTE > 2). The correlation increase after implementing the predictor is more significant for the group with extreme RTE.
(86 KB DOC)

Table S1 Protein Features Used in the Study Abbreviation and full description of all the protein features that were used in our study. We also checked the frequency of amino acids at the N and C terminus of the protein.
(52 KB DOC)

Table S2 The Correlation of the Predicted Protein Abundance of the Predictor with Real Protein Abundance, mRNA, tAI, and ER for Each GO Annotation Group Separately, and the Performances When Inferring a Different Predictor for Each Cellular Component GO (A–C) The correlation of the predicted protein abundance of our predictor with real protein abundance, mRNA, tAI, and ER for each GO annotation group separately. The last column includes the correlation of mRNA level with protein abundance for each GO group (blue, cases where the predictor improved the correlation with protein abundance; red, cases where the mRNA level has higher correlation with protein abundance).
(A) The results for the cellular component GO annotation groups.
(B) The results for the biological process GO annotation groups.
(C) The results for the molecular function GO annotation groups.
(D) The performances (correlation of predicted and real protein abundance) when inferring a different predictor for each cellular component GO annotation group. The average performances in this case are not better than the original predictor (one predictor for all the GO groups).
(209 KB DOC)

Table S3 Clustering (Sheet 1) and Bi-Clustering (Sheet 2) of the mRNA Gene Expression, from the Work of Sheehan et al. The list of genes in each cluster and bi-cluster is depicted together with the GO enrichment categories (for each of the ontologies: process, function, and component) of each cluster/bi-cluster. The score of each bi-cluster (by Expander) is depicted near the name of the bi-cluster (as mentioned by the authors of the pertaining Expander software used there, these scores are good only for comparing bi-clusters with the same size). The mean pattern of each bi-cluster and the index of conditions that are related to it (*x*-axis) appear near each bi-cluster.
(1.2 MB XLS)

Table S4 Clustering (Sheet 1) and Bi-Clustering (Sheet 2) of the Predicted Protein Abundance from the work of Sheehan et al. The list of genes in each cluster and bi-cluster is depicted together with the GO enrichment categories (for each of the ontologies: process, function, and component) of each cluster/bi-cluster. The score of each bi-cluster (by Expander) is depicted near the name of the bi-cluster (as mentioned by the authors of the pertaining Expander software used there, these scores are good only for comparing bi-clusters with the same size). The mean pattern of each bi-cluster and the index of conditions that are related to it (*x*-axis) appear near each bi-cluster.
(1.6 MB XLS)

Table S5 Clustering (Sheet 1) and Bi-Clustering (Sheet 2) of the mRNA Gene Expression from the Work of Tu et al. The list of genes in each cluster and bi-cluster is depicted together with the GO enrichment categories (for each of the ontologies: process, function, and component) of each cluster/bi-cluster. The score of each bi-cluster (by Expander) is depicted near the name of the bi-cluster (as mentioned by the authors of the pertaining Expander software used there, these scores are good only for comparing bi-clusters with the same size). The mean pattern of each bi-cluster and the index of conditions that are related to it (*x*-axis) appear near each bi-cluster.
(3 MB XLS)

Table S6 Clustering (Sheet 1) and Bi-Clustering (Sheet 2) of the Predicted Protein Abundance from the Work of Tu et al. The list of genes in each cluster and bi-cluster is depicted together with the GO enrichment categories (for each of the ontologies: process, function, and component) of each cluster/bi-cluster. The score of each bi-cluster (by Expander) is depicted near the name of the bi-cluster (as mentioned by the authors of the pertaining Expander software used there, these scores are good only for comparing bi-clusters with the same size). The mean pattern of each bi-cluster and the index of conditions that are related to it (*x*-axis) appear near each bi-cluster.
(2.1 MB XLS)

Table S7 Partial Correlations of Amino Acid Frequencies and Protein Abundance for All the Genes and for Genes with Low mRNA Levels and High Protein Abundance (A) Partial correlations of amino acid frequencies and protein abundance for all genes. The correlations for the amino acids alanine and valine are significant and positive, and the correlations for asparagine and serine are significant and negative.
(B) Partial correlations of the frequencies of amino acids and protein abundance for genes with low mRNA levels (lower 20%) and high protein abundance (top 20%). The correlations for the amino acids alanine and valine are positive but not significant (due to the low number of genes).
(13 KB XLS)

Table S8 Protein Abundance of the Various tRNA Synthetases and the Stechiometry of the Different Amino Acids Protein abundance of the various tRNA synthetases and the stechiometry of the different amino acids (downloaded from the work of Förster et al. [44]). Alanine and valine tRNA synthetases have high levels of protein abundance, and the amino acids exhibit a high concentration in the yeast cell. These factors also make the translation of alanine and valine more efficient. Data that do not appear in our dataset are denoted by ###.
(15 KB XLS)

Table S9 Genes with RTE > 2.5 Table includes the open reading frame (ORF), name, RTE, and description of each gene. Genes that are related with regulation are marked in blue. GO enrichments according to SGD for this group of genes appear below.
(27 KB XLS)

Table S10 Genes with RTE < 0.45 Table includes the ORF, name, RTE, and description of each gene. Genes that are related to regulation are marked in blue. GO enrichments according to SGD for this group of genes appear below.
(29 KB XLS)

Table S11 Subset of Genes That Exhibit Counteracting Regulatory Trends at the Transcriptional versus the Translational Levels (RTE < 1/1.5 and *m_SD_*/*m_YEPD_* > 1.5) Subset of genes that exhibit counteracting regulatory trends at the transcriptional versus the translational levels. Each gene in the set has RTE < 1/1.5 and *m_SD_*/*m_YEPD_* > 1.5. For each gene, the table includes its ORF ID, name, RTE, and the ratio between the mRNA levels in SD and YEPD.
(11 KB XLS)

Table S12 Subset of Genes That Exhibit Counteracting Regulatory Trends at the Transcriptional Versus the Translational Levels (RTE > 1/1.5 and *m_SD_*/*m_YEPD_* < 1.5) Subset of genes that exhibit counteracting regulatory trends at the transcriptional versus the translational levels. Each gene in the set has RTE > 1/1.5 and *m_SD_*/*m_YEPD_* < 1.5. For each gene, the table includes its ORF ID, name, RTE, and the ratio between the mRNA levels.
(32 KB XLS)

Table S13 RTE of Genes with Extreme TE (A) The RTE of the genes that were reported by Lu et al. as genes with high TE. The table includes the name, ORF, and RTE of each gene.
(B) The RTE of the 14 genes with extreme TE; in this case, the TE was calculated using the protein abundance of Ghaemmaghami et al. [2] and the mRNA levels of Holstege et al. [15] The table includes the name, ORF ID, RTE, TE, and TE rank (among all the genes) of each gene.
(12 KB XLS)

Text S1 Correlation Between Independent Measurements of Protein Abundance (24 KB DOC)

Text S2 Correlation Between mRNA Levels, Protein Abundance, and Predicted Protein Abundance between Interacting Proteins (25 KB DOC)

Text S3 Clustering and Bi-Clustering Predicted Protein Abundance (24 KB DOC)

Text S4 The Analysis of Steady-State and Transient Gene Expression Datasets (21 KB DOC)

Text S5 Clustering the Protein Abundance Levels Obtained from Random Predictors of Protein Abundance (25 KB DOC)

Text S6 Nonsignificant Improvement of the Predictor when Adding Amino Acid Frequencies (20 KB DOC)

Text S7 Variance in Protein Abundance for the Two Sets with Extreme RTEs (24 KB DOC)

Text S8 Supplementary Methods (25 KB DOC)

## Abbreviations

ARS: autonomously replicating sequence
CAI: codon adaptation index
ER: evolutionary rate
GO: Gene Ontology
ORF: open reading frame
RTE: relative translation efficiency
SD: synthetic dextrose
SVM: support vector machine
tAI: tRNA adaptation index
TE: translation efficiency
YEPD: yeast extract, peptone, and dextrose
