# Supplementary material for: Determinants of Protein Abundance and Translation Efficiency in S. cerevisiae
Source: PLoS Comput Biol. 2007 Dec 21;3(12):e248. doi: 10.1371/journal.pcbi.0030248 (PMC2230678; doi:10.1371/journal.pcbi.0030248)
Supplement: Figure S1 — (A) Variables that have significant correlation with protein abundance, TE, and RTE. (B) Variables with significant correlation with protein abundance given mRNA, given CAI, and given mRNA and CAI. The full names and the description of each variable appear in Table S1. The correlation with amino acid distribution at the C and N terminus was substantially less significant than the general correlations of amino acid distribution (it was not significant for most of the amino acids). (82 KB DOC) [file pcbi.0030248.sg001.doc]

**A.**

| Correlation with mRNA | | Correlation with Protein Abundance | | Correlation with Translation Efficiency | | Correlation with the change in Translation Efficiency | |
| --- | --- | --- | --- | --- | --- | --- | --- |
| Positive | Negative | Positive | Negative | Positive | Negative | Positive | Negative |
| tAI(0.69) | ER (-0.48) | tAI(0.63) | ER(-0.45) | GLU(0.17) | tAIi(-0.22) | PI(0.14) | Length(-0.27) |
| FOP (0.57) | CYS (-0.27) | CAI(0.61) | ASN(-0.29) | HL(0.13) | ER(-0.14) | HL(0.12) | MW(-0.27) |
| BIAS(0.56) | SER (-0.25) | FOP(0.61) | SER(-0.25) | ASP(0.12) | PI(-0.13) | LYS(0.11) | ASP(-0.08) |
| CAI (0.55) | LEU (-0.2) | BIAS(0.61) | MW(-0.15) | MW(0.11) | SER(-0.11) | ARG(0.07) | SER(-0.04) |
| ALA(0.37) | PHE (-0.2) | ALA (0.4) | Length(-0.14) | CAI(0.11) | MET(-0.05) | VAL(0.05) |  |
| GLY (0.29) | Aromaticity (-0.2) | HL(0.32) | LEU(-0.13) | Length(0.11) | THR(-0.04) |  |  |
| VAL (0.22) | ASN(-0.16) | VAL(0.28) | CYS(-0.11S) | BIAS(0.07) |  |  |  |
| GLU (0.13) | ILE(-0.13) | GLY(0.25) | HIS(-0.1) | LEU(0.07) |  |  |  |
| ASP (0.11) | PI(-0.1) | GRAVY(0.12) | MET(-0.09) | FOP(0.09) |  |  |  |
| LYS (0.11) | HIS(-0.1) |  | GLN(-0.09) | ALA(0.04) |  |  |  |
| GLN (0.06) | ARG(-0.08) |  | LYS(-0.07) | ILE(0.04) |  |  |  |
|  | TYR(-0.08) |  | ASP(-0.06) |  |  |  |  |
|  | GRAVY(-0.07) |  | Aromaticity (-0.06) |  |  |  |  |
|  | TRP(-0.05) |  | ILE(-0.06) |  |  |  |  |
|  | MET(-0.05) |  | PHE (-0.04) |  |  |  |  |
|  |  |  | TRP(-0.04) |  |  |  |  |
|  |  |  |  |  |  |  |  |

**B.**

| Correlation with protein abundance given mRNA levels | | Correlation with protein abundance given CAI | | Correlation with protein abundance given CAI and mRNA levels | |
| --- | --- | --- | --- | --- | --- |
| Positive | Negative | Positive | Negative | Positive | Negative |
| tAI (0.36) | ER(-0.28) | tAI (0.13) | ER(-0.26) | tAI (0.1) | ER(-0.27) |
| CAI (0.32) | SER(-0.17) | mRNA(0.36) | MW(-0.2) | HL(0.25) | MW(-0.18) |
| FOP(0.31) | PI(-0.13) | HL(0.26) | Length(-0.19) | ALA(0.2) | CAI(-0.18) |
| BIAS(0.29) | ASP(-0.1) | ALA(0.21) | SER(-0.17) | VAL(0.16) | Length(-0.17) |
| HL(0.22) | ASN(-0.08) | VAL(0.17) | ASN(-0.16) | GLY(0.14) | SER(-0.16) |
| ALA(0.15) | CYS(-0.06) | GLY(0.16) | PI(-0.08) | BIAS(0.12) | ASN(-0.14) |
| GLU(0.11) | THR(-0.06) | BIAS(0.15) | HIS(-0.08) | FOP(0.11) | PI(-0.08) |
| VAL(0.11) | HIS(-0.05) | FOP(0.14) | PHE(-0.06) | GRAVY(0.1) | HIS(-0.08) |
| GLY(0.07) | PRO(-0.05) | GRAVY(0.1) | Aromaticity (-0.06) | GLU(0.08) | ASN(-0.07) |
| LYS(0.05) | TRP(-0.04) | GLU(0.08) | THR(-0.04) |  | PHE(-0.06) |
| GRAVY(0.03) | Aromaticity (-0.04) |  | TYR(-0.04) |  | Aromaticity (-0.06) |
|  | Arg(-0.03) |  | LYS(-0.04) |  | THR(-0.04) |
|  |  |  | CYS(-0.04) |  | TYR(-0.04) |
|  |  |  | PRO(-0.04) |  | CYS(-0.04) |
|  |  |  |  |  | PRO(-0.04) |

Figure S1 A. Variables that have significant correlation with protein abundance, TE, and RTE. B. Variables with significant correlation with protein abundance given mRNA, given CAI, and given mRNA and CAI. The full names and the description of each variable appear in table S1 of the supplementary material. The correlation with amino acid distribution at the C and N terminus was substantially less significant than the general correlations of amino acid distribution (it was not significant for most of the amino acids).
