# Supplementary material for: Determinants of Protein Abundance and Translation Efficiency in S. cerevisiae
Source: PLoS Comput Biol. 2007 Dec 21;3(12):e248. doi: 10.1371/journal.pcbi.0030248 (PMC2230678; doi:10.1371/journal.pcbi.0030248)
Supplement: Figure S2 — (53 KB DOC) [file pcbi.0030248.sg002.doc]

Figure S2: Partial correlations of amino acids' frequencies and protein abundance after removing the effect of CAI.
