# Supplementary material for: Determinants of Protein Abundance and Translation Efficiency in S. cerevisiae
Source: PLoS Comput Biol. 2007 Dec 21;3(12):e248. doi: 10.1371/journal.pcbi.0030248 (PMC2230678; doi:10.1371/journal.pcbi.0030248)
Supplement: Figure S3 — The average RTE of each GO annotation group for the three ontologies (molecular function, cellular component, and biological process). (71 KB DOC) [file pcbi.0030248.sg003.doc]

Figure S3. The average RTE of each GO annotation group for the three ontologies (molecular function, cellular component, and biological process).
