# Supplementary material for: Determinants of Protein Abundance and Translation Efficiency in S. cerevisiae
Source: PLoS Comput Biol. 2007 Dec 21;3(12):e248. doi: 10.1371/journal.pcbi.0030248 (PMC2230678; doi:10.1371/journal.pcbi.0030248)
Supplement: Figure S4 — (A) mRNA levels and protein abundance of genes with RTE > 2.5 (blue), RTE < 0.45 (red), and the rest of the genes (yellow) in YEPD. (B) mRNA levels and protein abundance of genes with RTE > 2.5 (blue), RTE < 0.45 (red), and the rest of the genes (yellow) in SD. (C) mRNA ratio (mSD/mYEPD) levels and protein abundance ratio (pSD/pYEPD) of genes with RTE > 2.5 (blue), RTE < 0.45 (red), and the rest of the genes (yellow). (D) Correlation with protein abundance of mRNA and predicted protein abundance for genes with modest RTE (0.5 < RTE < 2), and for genes with extreme RTE (RTE < 0.5 and RTE > 2). The correlation increase after implementing the predictor is more significant for the group with extreme RTE. (86 KB DOC) [file pcbi.0030248.sg004.doc]

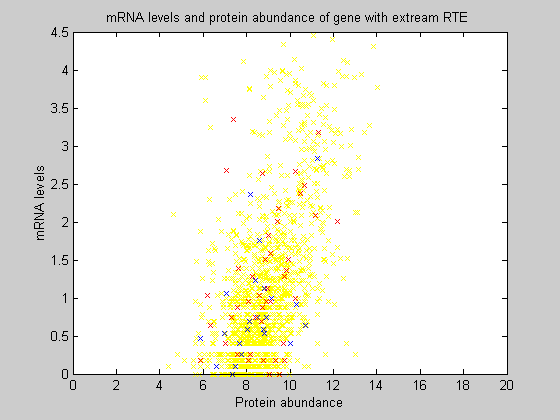


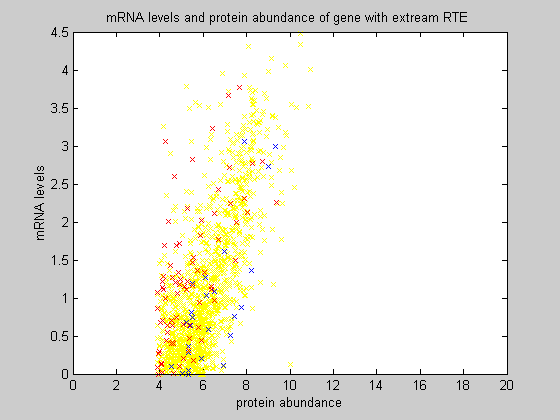


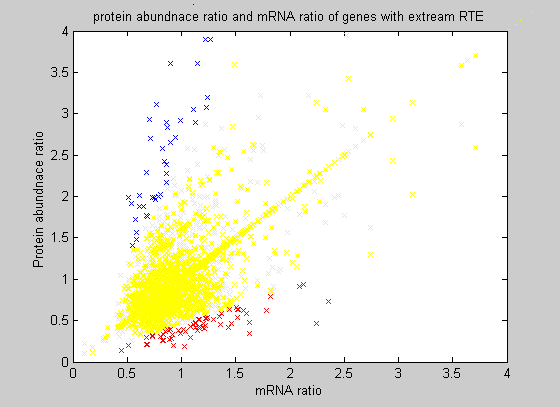


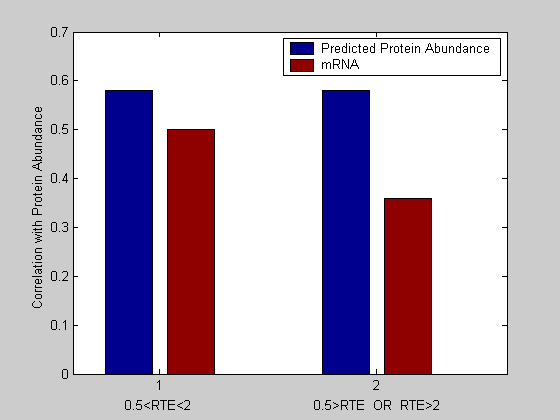


Figure S4. A. mRNA levels and protein abundance of genes with RTE > 2.5 (blue), RTE<0.45 (red), and the rest of the genes (yellow) in YEPD. B. mRNA levels and protein abundance of genes with RTE > 2.5 (blue), RTE<0.45 (red), and the rest of the genes (yellow) in SD. C. mRNA ratio (mSD/mYEPD) levels and protein abundance ratio (PSD/PYEPD) of genes with RTE > 2.5 (blue), RTE<0.45 (red), and the rest of the genes (yellow). D. Correlation with protein abundance of mRNA and predicted protein abundance for genes with modest RTE (0.5<RTE<2), and for genes with extreme RTE (RTE < 0.5 and RTE>2). The correlation increase after implementing the predictor is more significant for the group with extreme RTE.
