# Supplementary material for: Determinants of Protein Abundance and Translation Efficiency in S. cerevisiae
Source: PLoS Comput Biol. 2007 Dec 21;3(12):e248. doi: 10.1371/journal.pcbi.0030248 (PMC2230678; doi:10.1371/journal.pcbi.0030248)
Supplement: Table S1 — Abbreviation and full description of all the protein features that were used in our study. We also checked the frequency of amino acids at the N and C terminus of the protein. (52 KB DOC) [file pcbi.0030248.st001.doc]

| Index | Abbreviation | Full description |
| --- | --- | --- |
| 1 | MW | Molecular weight |
| 2 | PI | Net charge of protein in aqueous solution |
| 3 | CAI | Codon Adaptation Index |
| 4 | PL | Protein length |
| 5 | CB | Codon bias |
| 6 | ALA | Frequency of the amino acid Alanine in the protein |
| 7 | ARG | Frequency of the amino acid Arginine in the protein |
| 8 | ASN | Frequency of the amino acid Asparagine in the protein |
| 9 | ASP | Frequency of the amino acid Aspartic acid in the protein |
| 10 | CYS | Frequency of the amino acid Cysteine in the protein |
| 11 | GLN | Frequency of the amino acid Glutamine in the protein |
| 12 | GLU | Frequency of the amino acid Glutamic acid in the protein |
| 13 | GLY | Frequency of the amino acid Glycine in the protein |
| 14 | HIS | Frequency of the amino acid Histidine in the protein |
| 15 | ILE | Frequency of the amino acid Isoleucine in the protein |
| 16 | LEU | Frequency of the amino acid Leucine in the protein |
| 17 | LYS | Frequency of the amino acid Lysine in the protein |
| 18 | MET | Frequency of the amino acid Methionine in the protein |
| 19 | PHE | Frequency of the amino acid Phenylalanine in the protein |
| 20 | PRO | Frequency of the amino acid Proline in the protein |
| 21 | SER | Frequency of the amino acid Serine in the protein |
| 22 | THR | Frequency of the amino acid Threonine in the protein |
| 23 | TRP | Frequency of the amino acid Tryptophan in the protein |
| 24 | TYR | Frequency of the amino acid Tyrosine in the protein |
| 25 | VAL | Frequency of the amino acid Valine in the protein |
| 26 | FOP | Frequency of optimal codons |
| 27 | GRAV | Gravy, hydropathicity of Protein |
| 28 | AROM | Aromaticity (Frequency of aromatic amino acids: Phe, Tyr, Trp) |
| 29 | HL | Half life |
| 30 | ER | Evolutionary rate |
| 31 | TE | Translation efficiency |
| 32 | tAI | tRNA adaptation index |

Table S1. Abbreviation and full description of all the proteins' features that were used in our study. We also checked the frequency of amino acids at the N and C terminus of the protein.
