# Supplementary material for: Determinants of Protein Abundance and Translation Efficiency in S. cerevisiae
Source: PLoS Comput Biol. 2007 Dec 21;3(12):e248. doi: 10.1371/journal.pcbi.0030248 (PMC2230678; doi:10.1371/journal.pcbi.0030248)
Supplement: Table S2 — (A–C) The correlation of the predicted protein abundance of our predictor with real protein abundance, mRNA, tAI, and ER for each GO annotation group separately. The last column includes the correlation of mRNA level with protein abundance for each GO group (blue, cases where the predictor improved the correlation with protein abundance; red, cases where the mRNA level has higher correlation with protein abundance). (A) The results for the cellular component GO annotation groups. (B) The results for the biological process GO annotation groups. (C) The results for the molecular function GO annotation groups. (D) The performances (correlation of predicted and real protein abundance) when inferring a different predictor for each cellular component GO annotation group. The average performances in this case are not better than the original predictor (one predictor for all the GO groups). (209 KB DOC) [file pcbi.0030248.st002.doc]

**A.**

| mRNA vs  Protein abundance | tAI | ER | Protein abundance | mRNA | N^O^  of Genes | GO group | Index |
| --- | --- | --- | --- | --- | --- | --- | --- |
| 0.45 | 0.76 | -0.7 | 0.52 | 0.86 | 61 | Bud | 1 |
| 0.6 | 0.89 | -0.65 | 0.64 | 0.87 | 39 | Cell_cortex | 2 |
| 0.31 | 0.96 | -0.32 | 0.36 | 0.94 | 15 | Cell_wall | 3 |
| 0.52 | 0.8 | -0.72 | 0.51 | 0.79 | 88 | Chromosome | 4 |
| 0.52 | 0.88 | -0.68 | 0.64 | 0.85 | 533 | Cytoplasm | 5 |
| 0.57 | 0.82 | -0.65 | 0.65 | 0.92 | 41 | Cytoplasmic membrane bound vesicle | 6 |
| 0.59 | 0.78 | -0.6 | 0.5 | 0.86 | 75 | Cytoskeleton | 7 |
| 0.45 | 0.89 | -0.7 | 0.63 | 0.85 | 121 | Endomembrane system | 8 |
| 0.47 | 0.87 | -0.69 | 0.6 | 0.84 | 137 | Endoplasmic reticulum | 9 |
| 0.45 | 0.8 | -0.61 | 0.61 | 0.86 | 74 | Golgi apparatus | 10 |
| 0.46 | 0.87 | -0.67 | 0.6 | 0.87 | 381 | Membrane | 11 |
| 0.59 | 0.86 | -0.77 | 0.73 | 0.86 | 33 | Membrane fraction | 12 |
| 0.33 | 0.6 | -0.59 | 0.18 | 0.77 | 22 | Microtubule organizing center | 13 |
| 0.56 | 0.9 | -0.7 | 0.69 | 0.89 | 123 | Mitochondrial envelope | 14 |
| 0.52 | 0.87 | -0.7 | 0.62 | 0.86 | 477 | Mitochondrion | 15 |
| 0.52 | 0.94 | -0.61 | 0.6 | 0.87 | 90 | Nucleolus | 16 |
| 0.57 | 0.85 | -0.64 | 0.64 | 0.85 | 536 | Nucleus | 17 |
| 0.36 | 0.8 | -0.77 | 0.72 | 0.56 | 21 | Peroxisome | 18 |
| 0.39 | 0.8 | -0.7 | 0.5 | 0.88 | 65 | Plasma membrane | 19 |
| 0.49 | 0.92 | -0.7 | 0.59 | 0.88 | 83 | Ribosome | 20 |
| 0.39 | 0.79 | -0.73 | 0.44 | 0.78 | 60 | Site of polarized growth | 21 |
| 0.47 | 0.84 | -0.61 | 0.49 | 0.86 | 68 | Vacuole | 22 |

**B.**

| mRNA vs  Protein abundance | tAI | ER | Protein abundnace | mRNA | N^O^  of Genes | GO group | Index |
| --- | --- | --- | --- | --- | --- | --- | --- |
| 0.53 | 0.8 | -0.64 | 0.6 | 0.82 | 211 | DNA Metabolism | 1 |
| 0.74 | 0.94 | -0.65 | 0.84 | 0.88 | 73 | Carbohydrate metabolism | 2 |
| 0.5 | 0.86 | -0.68 | 0.57 | 0.87 | 32 | Cell budding | 3 |
| 0.54 | 0.73 | -0.6 | 0.66 | 0.87 | 143 | Cell cycle | 4 |
| 0.53 | 0.88 | -0.73 | 0.59 | 0.9 | 48 | Cell homeostasis | 5 |
| 0.39 | 0.92 | -0.33 | 0.48 | 0.88 | 50 | Cell wall organization and biogenesis | 6 |
| 0.64 | 0.92 | -0.7 | 0.8 | 0.84 | 35 | Cellular respiration | 7 |
| 0.35 | 0.72 | -0.75 | 0.55 | 0.77 | 36 | Conjugation | 8 |
| 0.52 | 0.85 | -0.73 | 0.49 | 0.86 | 36 | Cytokinesis | 9 |
| 0.48 | 0.83 | -0.52 | 0.49 | 0.86 | 80 | Cytoskeletonorganization  and biogenesis | 10 |
| 0.64 | 0.93 | -0.72 | 0.69 | 0.89 | 78 | Amino acid and derivative metabolism | 11 |
| -0.01 | 0.43 | -0.03 | 0.11 | 0.7 | 13 | Electron transport | 12 |
| 0.66 | 0.92 | -0.69 | 0.76 | 0.88 | 87 | Generation of precursormetabolites and energy | 13 |
| 0.62 | 0.83 | -0.62 | 0.71 | 0.9 | 89 | Lipid metabolism: | 14 |
| 0.51 | 0.78 | -0.48 | 0.52 | 0.93 | 38 | Meiosis | 15 |
| 0.35 | 0.78 | -0.7 | 0.43 | 0.76 | 34 | Membrane organization and biogenesis | 16 |
| 0.60 | 0.82 | -0.73 | 0.61 | 0.85 | 103 | Morphogenesis | 17 |
| 0.23 | 0.76 | -0.74 | 0.63 | 0.6 | 25 | Nuclear organization and biogenesis | 18 |
| 0.58 | 0.86 | -0.64 | 0.65 | 0.87 | 516 | Organelle organization and biogenesis: | 19 |
| 0.59 | 0.91 | -0.72 | 0.66 | 0.9 | 179 | Protein biosynthesis | 20 |
| 0.68 | 0.9 | -0.78 | 0.71 | 0.91 | 78 | Protein catabolism | 21 |
| 0.58 | 0.85 | -0.67 | 0.65 | 0.88 | 241 | Protein modification | 22 |
| 0.59 | 0.92 | -0.76 | 0.7 | 0.85 | 33 | Pseudohyphal growth | 23 |
| 0.62 | 0.89 | -0.72 | 0.73 | 0.87 | 169 | Response to stress | 24 |
| 0.50 | 0.94 | -0.63 | 0.65 | 0.82 | 147 | Ribosome biogenesis and assembly | 25 |
| 0.59 | 0.86 | -0.64 | 0.64 | 0.87 | 222 | RNA Metabolism | 26 |
| 0.63 | 0.82 | -0.7 | 0.7 | 0.84 | 69 | Signal transduction | 27 |
| 0.78 | 0.86 | -0.71 | 0.75 | 0.94 | 27 | Sporulation | 28 |
| 0.48 | 0.75 | -0.67 | 0.52 | 0.83 | 228 | Transcription | 29 |
| 0.49 | 0.85 | -0.68 | 0.62 | 0.86 | 332 | Transport | 30 |
| 0.53 | 0.82 | -0.65 | 0.65 | 0.89 | 112 | Vesicle mediated transport | 31 |
| 0.65 | 0.87 | -0.63 | 0.6 | 0.83 | 26 | Vitamin metabolism | 32 |

**C.**

| mRNA vs  Protein abundance | tAI | ER | Protein abundnace | mRNA | N^O^  of Genes | GO group | Index |
| --- | --- | --- | --- | --- | --- | --- | --- |
| 0.61 | 0.83 | -0.67 | 0.66 | 0.87 | 82 | DNA binding | 1 |
| 0.57 | 0.82 | -0.64 | 0.66 | 0.88 | 78 | Enzyme regulator activity | 2 |
| 0.32 | 0.86 | -0.62 | 0.51 | 0.85 | 30 | Helicase activity | 3 |
| 0.63 | 0.85 | -0.66 | 0.67 | 0.87 | 291 | Hydrolase activity | 4 |
| 0.17 | 0.95 | -0.68 | 0.19 | 0.87 | 19 | Isomerase activity | 5 |
| 0.62 | 0.88 | -0.77 | 0.69 | 0.87 | 40 | Ligase activity | 6 |
| 0.78 | 0.93 | -0.83 | 0.81 | 0.94 | 35 | Lyase activity | 7 |
| 0.17 | 0.93 | -0.46 | -0.67 | -0.5 | 7 | Motor activity | 8 |
| 0.47 | 0.81 | -0.56 | 0.44 | 0.81 | 32 | Nucleotidyltransferase activity | 9 |
| 0.51 | 0.88 | -0.62 | 0.63 | 0.86 | 106 | Oxidoreductase activity | 10 |
| 0.61 | 0.81 | -0.74 | 0.6 | 0.91 | 48 | Peptidase activity | 11 |
| 0.36 | 0.73 | -0.37 | 0.63 | 0.75 | 22 | Phosphoprotein phosphataseactivity | 12 |
| 0.53 | 0.84 | -0.69 | 0.66 | 0.84 | 203 | Protein binding | 13 |
| 0.48 | 0.87 | -0.74 | 0.57 | 0.87 | 37 | Protein kinase activity | 14 |
| 0.62 | 0.88 | -0.68 | 0.68 | 0.86 | 111 | RNA binding | 15 |
| 0.2 | 0.76 | -0.86 | 0.31 | 0.82 | 16 | Signal transducer activity | 16 |
| 0.37 | 0.83 | -0.7 | 0.5 | 0.8 | 110 | Structural molecule activity | 17 |
| 0.34 | 0.71 | -0.63 | 0.45 | 0.77 | 130 | Transcription regulator activity | 18 |
| 0.59 | 0.87 | -0.67 | 0.67 | 0.88 | 259 | Transferase activity | 19 |
| 0.57 | 0.96 | -0.67 | 0.64 | 0.94 | 25 | Translation regulator activity | 20 |
| 0.49 | 0.93 | -0.73 | 0.59 | 0.91 | 98 | Transporter activity | 21 |

**D.**

| Correlation | N^O^  of Genes | GO group | Index |
| --- | --- | --- | --- |
| 0.5852 | 61 | Bud | 1 |
| 0.6439 | 39 | Cell_cortex | 2 |
| 0.0445 | 15 | Cell_wall | 3 |
| 0.5822 | 88 | Chromosome | 4 |
| 0.6371 | 533 | Cytoplasm | 5 |
| 0.6949 | 41 | Cytoplasmic membrane bound vesicle | 6 |
| 0.5529 | 75 | Cytoskeleton | 7 |
| 0.6735 | 121 | Endomembrane system | 8 |
| 0.6254 | 137 | Endoplasmic reticulum | 9 |
| 0.6484 | 74 | Golgi apparatus | 10 |
| 0.6220 | 381 | Membrane | 11 |
| 0.7781 | 33 | Membrane fraction | 12 |
| -0.4036 | 22 | Microtubule organizing center | 13 |
| 0.7133 | 123 | Mitochondrial envelope | 14 |
| 0.6172 | 477 | Mitochondrion | 15 |
| 0.6374 | 90 | Nucleolus | 16 |
| 0.6493 | 536 | Nucleus | 17 |
| 0.3214 | 21 | Peroxisome | 18 |
| 0.5581 | 65 | Plasma membrane | 19 |
| 0.6043 | 83 | Ribosome | 20 |
| 0.5321 | 60 | Site of polarized growth | 21 |
| 0.5606 | 68 | Vacuole | 22 |

Table S2: A-C: The correlation of the predicted protein abundance of our predictor with real protein abundance, mRNA, tAI, and ER for each GO annotation group separately. The last column includes the correlation of mRNA level with protein abundance for each GO group (in blue – cases where the predictor improved the correlation with protein abundance, in red – cases where the mRNA level has higher correlation with protein abundance) .

A. The results for the cellular component GO annotation groups. B. The results for the biological process GO annotation groups. C. The results for the molecular function GO annotation groups. D. The performances (correlation of predicted and real protein abundance) when inferring a different predictor for each cellular component GO annotation group. The average performances in this case are not better than the original predictor (one predictor for all the GO groups).
