# Supplementary material for: Determinants of Protein Abundance and Translation Efficiency in S. cerevisiae
Source: PLoS Comput Biol. 2007 Dec 21;3(12):e248. doi: 10.1371/journal.pcbi.0030248 (PMC2230678; doi:10.1371/journal.pcbi.0030248)
Supplement: Text S1 — (24 KB DOC) [file pcbi.0030248.sd001.doc]

**Note 1: Correlation between independent measurements of protein aboundance.**

In general the correlation between two proteomic datasets generated by two techniques and in different labs is quite high, varying between r_s_ = 0.6 to r_s_ = 0.8. For example (see Lu *et al.* 2006): the APEX vs. Western blot gives r_s_ = 0.61, APEX vs. flow cytometry gives r_s_ = 0.69, APEX vs. 2D gel gives r_s_ = 0.8, APEX vs. GFP fusion protein quantities gives 0.69, (Newman *et al*. 2006): flow cytometry vs Western blot gives r_s_ = 0.8. The correlation between two major (largest) protein abundance measurements (Ghaemmaghami *et al.* 2003 vs Newman *et al*. 2006) in the same condition (YEPD) is r_s_ = 0.61, p-value < 10^-50^ (2268 genes).

When using the same technology and lab the correlations become even higher. For APEX (Lu *et al.* 2006) it is r_s_ = 0.98, for flow cytometry (Newman *et al*. 2006) it is r_s_ = 0.99, for Western blot (Newman *et al*. 2006) it is r_s_ = 0.77, and for mass spectrometry (Schmidt *et al*. 2007) r_s_ = 0.85.
