# Supplementary material for: Determinants of Protein Abundance and Translation Efficiency in S. cerevisiae
Source: PLoS Comput Biol. 2007 Dec 21;3(12):e248. doi: 10.1371/journal.pcbi.0030248 (PMC2230678; doi:10.1371/journal.pcbi.0030248)
Supplement: Text S2 — (25 KB DOC) [file pcbi.0030248.sd002.doc]

**Note 2: *The correlation between mRNA levels, protein abundance and predicted protein abundance between interacting proteins***

In addition to the analysis of the coherence of proteins within known complexes (see Methods), we examined if protein abundance levels (both actual and predicted) are more coherent for pairs of adjacent proteins in the protein-protein interaction network than for random protein pairs. The results showed a significant Spearman correlation between the measured protein abundance of pairs of adjacent proteins (0.18 and 0.15 in YEPD and SD respectively with p-values 10^-16^ and 10^-15^ respectively). Similar values were obtained when using the predicted protein levels (0.175 and 0.144 in YEPD and SD respectively with p-values 10^-16^ and 10^-15^ respectively). The Spearman correlation between mRNA levels of adjacent proteins is smaller but significant (0.14 and 0.13 in YEPD and SD, with p-values of 10^-15^ and 10^-14^ respectively). The Spearman correlation between the protein abundance of non-adjacent proteins is vanishing low in all cases.
