# Supplementary material for: Determinants of Protein Abundance and Translation Efficiency in S. cerevisiae
Source: PLoS Comput Biol. 2007 Dec 21;3(12):e248. doi: 10.1371/journal.pcbi.0030248 (PMC2230678; doi:10.1371/journal.pcbi.0030248)
Supplement: Text S3 — (24 KB DOC) [file pcbi.0030248.sd003.doc]

**Note 3: Clustering and bi-clustering predicted protein abundance.**

There are many definitions for pair-wise distances between vectors (or in particular the expression levels of genes). If the pair-wise distance used is, e.g, the correlation of the log of the vectors, then, indeed, the predictor cannot change the pair wise distances. However, in many cases in the literature other distance metrics are used, which are not invariant to the kind of transformations induced by our protein abundance predictor. Specifically, we used the Click algorithm (Sharan and Shamir 2000) for clustering, and the SAMBA (Tanay *et al.* 2002) algorithm for bi-clustering. The pair wise distances in Click are dot-products. SAMBA uses a probabilistic graphical model for defining a significant bi-cluster which is related (among other factors) to the average expression of each gene and the variation (ratio) from this average. In these two cases using a protein abundance predictor can change (and indeed changes) the resulting clustering/bi-clustering.
