# Supplementary material for: Determinants of Protein Abundance and Translation Efficiency in S. cerevisiae
Source: PLoS Comput Biol. 2007 Dec 21;3(12):e248. doi: 10.1371/journal.pcbi.0030248 (PMC2230678; doi:10.1371/journal.pcbi.0030248)
Supplement: Text S4 — (21 KB DOC) [file pcbi.0030248.sd004.doc]

**Note 4: The analysis of steady-state and transient gene expression datasets.**

The dynamic nature of transcriptional, translational, and degradation processes, may require significant time to reach equilibrium in the cell. During the transient phase, mRNA and its corresponding protein concentration may even be anti correlated. However, at or near equilibrium, the two are likely to be more correlated. We analyzed two gene expression datasets, one from each type.

The first includes gene expression measurements in a system that is near to equilibrium - yeast under low-shear modeled microgravity and its control group, where in both cases the yeast was incubated for 5-25 generations. The second includes gene expression measurements in a system that is in a transient state. It includes samples of gene expression of yeast (every 25 mins) that was grown in nutrient-limited conditions and thus exhibit robust, highly periodic cycles in the form of respiratory bursts.

The predictor has better performance on the first dataset (a system that is near to equilibrium), but two datasets are obviously insufficent for drawing general conclusions about the performance of the predictor in steady-state vs transients conditions.
