# Supplementary material for: Determinants of Protein Abundance and Translation Efficiency in S. cerevisiae
Source: PLoS Comput Biol. 2007 Dec 21;3(12):e248. doi: 10.1371/journal.pcbi.0030248 (PMC2230678; doi:10.1371/journal.pcbi.0030248)
Supplement: Text S5 — (25 KB DOC) [file pcbi.0030248.sd005.doc]

**Note 5: Clustering the protein abundance levels obtained from random predictors of protein abundance.**

To test the statistical significance of the clusters obtained by our predictor we performed the following experiment:

Let **T** denote the maximum tAI in our dataset, and let **E** denote the maximum evolutionary rate in our dataset. Instead of using our predictor, we examined the performance of a random predictor that used instead of the tAI values a random variable with uniform distribution U[0,T], and instead of pertaining evolutionary rate it used a random variable with a uniform distribution U[0,E]. We constructed a 100 such random predictors, and applied the same clustering procedures on their resulting `predicted' protein abundance levels.. The results were clearly worse than those obtained both with the mRNA levels and our predicted protein abundance values. For example, for the first dataset (yeast under low-shear modeled microgravity), about 50% of the clusters obtained from the random predictions included no enrichments, and the top enrichments (GO process ontology) in the resulting clusters were in the order of 10^-4^-10^-6^. In comparison, in the case of the mRNA measurements, the top enrichments were in the order of 10^-13^-10^-38^ and 57% of the clusters were enriched. In the case of the predicted protein abundance levels, 85% of the clusters were enriched, with top enrichments in the order of 10^-13^-10^-38^, showing the clear superiority of the latter over both the random and mRNA clusters.
