# Supplementary material for: Determinants of Protein Abundance and Translation Efficiency in S. cerevisiae
Source: PLoS Comput Biol. 2007 Dec 21;3(12):e248. doi: 10.1371/journal.pcbi.0030248 (PMC2230678; doi:10.1371/journal.pcbi.0030248)
Supplement: Text S6 — (20 KB DOC) [file pcbi.0030248.sd006.doc]

**Note 6: Non significant improvement of the predictor when adding amino acids frequencies**

We have tried to incorporate the amino acid frequencies in our prediction model (see supplementary Table S1). However the improvement when adding amino acids to the predictor was marginal and insignificant (the correlation remained very close to 0.63).  Note that while the linear predictor we used (giving the best performance on the first three leading variables) implicitly relies on normality assumptions, the partial correlations with amino acids are measured via the Spearman rank correlation (which does not entail a normally distributed data). Thus it may well be that the predictor used, while doing very well on the first leading variables, may fail to capture all the predictability potential gleaned in the subsequent input variables. However, given its simplicity and superior performance with the first leading variables, we choose to adhere to the choice of the predictor presented in the main text.
