# Supplementary material for: Determinants of Protein Abundance and Translation Efficiency in S. cerevisiae
Source: PLoS Comput Biol. 2007 Dec 21;3(12):e248. doi: 10.1371/journal.pcbi.0030248 (PMC2230678; doi:10.1371/journal.pcbi.0030248)
Supplement: Text S7 — (24 KB DOC) [file pcbi.0030248.sd007.doc]

**Note 7: *Variance in protein abundance for the two sets with extreme RTEs***

For evaluating the variance of protein abundance in the two groups with extreme RTEs, we used the protein abundance data of (Newman *et al*. 2006). The average variance for all the genes in the dataset is 159.85 and 120.41 proteins' copies per cell in YEPD and SD respectively; the average variance for the first set of genes (genes with RTE > 2.5) is 158.77 and 115.00 proteins' copies per cell for YEPD and SD respectively; the average variance for the second set of genes (genes with RTE < 0.45) is 358.13 and 165.699 proteins' copies per cell for YEPD and SD respectively.

The variance in the first group is markedly smaller than that of the second one, suggesting that it is more tightly regulated by environmental conditions.
