# Supplementary material for: Determinants of Protein Abundance and Translation Efficiency in S. cerevisiae
Source: PLoS Comput Biol. 2007 Dec 21;3(12):e248. doi: 10.1371/journal.pcbi.0030248 (PMC2230678; doi:10.1371/journal.pcbi.0030248)
Supplement: Text S8 — (25 KB DOC) [file pcbi.0030248.sd008.doc]

**Supplementary Methods for the paper: "Determinants of Protein Abundance and Translation Efficiency in Yeast"**

*Tamir Tuller, Martin Kupiec & Eytan Ruppin*

***Distance of genes from an origin of replication***

Data about the positions of origins of replication (ARS) and the beginning/end of each gene was taken from [20]. The distance of a gene from an origin of replication is defined as the minimal distance of one of the gene's ends from an origin of replication. If there is an origin of replication between the two ends of a gene, the distance is defined as zero.

***Coherency of expression levels of proteins that are part of the same complex after removing the effect of mRNA***

Let *u^p^* and *v^p^* denote two vectors of protein abundance or predicted protein abundance measurements generated as described in the Methods, and let *u^m^* and *v^m^* denote the corresponding vectors of mRNA measurements; let C(x,y|z) and P(x,y|z) denote the partial Spearman correlation and corresponding p-value of x and y given z.

For evaluating the correlation between protein abundance, and predicted protein abundance after removing the effect of mRNA level we used (C(*u^p^*, *v^p^* | *u^m^*) + C(*u^p^*, *v^p^* | *v^m^*) )/2 for the partial correlation, and (P(*u^p^*, *v^p^* | *u^m^*) + P(*u^p^*, *v^p^* | *v^m^*) )/2 for a corresponding p-value (in all cases C(*u^p^*, *v^p^* | *u^m^*) was similar to C(*u^p^*, *v^p^* | *v^m^*), and P(*u^p^*, *v^p^* | *u^m^*) was similar to P(*u^p^*, *v^p^* | *v^m^*).
